# To Our Patrons

**Published:** 1857-08

**Authors:** 


### TO OUR PATRONS.

With this number will close the second volume of our Journal and the connection of the Faculty of the Atlanta Medical College with it as publishers, the Journal having been transferred to G. P. Eddy & Co.

We shall however continue to feel an undiminished interest in the success of the Journal, and as *Editors* will continue to make every exertion not only to sustain, but to advance its standing. All communications referring to the third volume must be addressed to G. P. Eddy & Co., publishers of Atlanta Medical Journal. Those having reference to the previous volumes, to J. G. Westmoreland, to whom all dues on account of these must be paid; subscriptions to the third volume, being paid of course to the new publishers, for whom we earnestly ask the interest of our friends to increase the subscription list.

We are requested to say that it is very important that the published terms—payment in advance—should be rigidly observed.

